# Use of cryotherapy to treat obstructing papilloma of an accessory tracheal bronchus: case report

**DOI:** 10.1186/s13019-022-01977-6

**Published:** 2022-10-22

**Authors:** Alexandra E. Adams, Federico A. Steiner

**Affiliations:** 1 Department of Surgery, Morristown Medical Center, 100 Madison Ave, Morristown, NJ 07960 USA; 2 Division of Cardiothoracic Surgery, Morristown Medical Center, 100 Madison Ave, Morristown, NJ 07960 USA

**Keywords:** Bronchoscopy/bronchus, Cryotherapy, Tracheal, Tumor, Case report

## Abstract

**Background:**

Tracheal papillomatosis is a relatively rare condition with limited data on successful treatment modalities. To our knowledge, this is the first report to describe a papilloma arising from an accessory bronchus. Furthermore, this case report demonstrates successful treatment with clinical and patient-centered improvements after use of Spray Cryotherapy.

**Case presentation:**

A 71-year-old woman presented with one year history of recurrent fevers and intermittent hemoptysis. Imaging and video bronchoscopy revealed an obstructing papilloma of an accessory tracheal bronchus to the right upper lobe. She was treated with debridement followed by multiple cryotherapy treatments resulting in complete clinical and radiographic resolution of her post-obstructive pneumonia.

**Conclusions:**

This case report not only supports existing literature on the use of cryotherapy for airway diseases but also presents a unique form of obstructing papilloma confined to an accessory bronchus, the only report of its kind based on extensive literature review.

## Introduction

Tracheal papillomatosis is characterized by papillomatous growth of the bronchial epithelium associated with Human Papilloma Virus (HPV). Occurring in either the larynx or trachea, this is a relatively rare condition with bimodal expression in children and adults in their 5th–6th decade [1]. In the adult population, incidence is estimated at about 18 in 1 million and, though low, there is a risk for transformation to squamous cell carcinoma (2%) [1]^.^ Symptoms of tracheal papillomatosis are largely non-specific, ranging from dyspnea to hemoptysis. Several treatment modalities have been suggested in the literature including probe-directed cryotherapy, CO_2_ laser ablation, interferon-a injection, and surgical excision [1–4]. In this case, cryotherapy was elected as the preferred treatment modality. Given the involvement of an accessory bronchus, surgical resection would have been complex requiring lobectomy and tracheoplasty to obtain an R0 resection. In contrast, cryotherapy is a well-tolerated lung sparing outpatient procedure that can preserve quality of life, especially in patients with aberrant anatomy or compromised lung function. We elected to use the novel approach of trufreeze® Spray Cryotherapy ablation technology (Steris, Mentor, OH, US). This method of cryotherapy differs from traditional probe application, where direct contact with the probe is required and tissue removal is often the result. Spray cryotherapy allows for even application of cryogen without requiring direct contact with the probe, resulting in evenly distributed tissue damage and cell death. The resulting even distribution of treatment is desirable in dysplastic tissue. Furthermore, this patient’s anatomy lent itself well to this treatment modality since the angulation of the video bronchoscope accommodates the flexible cryo-spray catheter, making the lesion easily accessible. This patient had an accessory tracheal bronchus, a congenital tracheobronchial anomaly defined by a bronchus that arises superior to the carina with reported incidence of 0.2% [6]. To our knowledge, this is the first report to describe a papilloma arising from an accessory bronchus.

### Case report

**Fig. 1:**
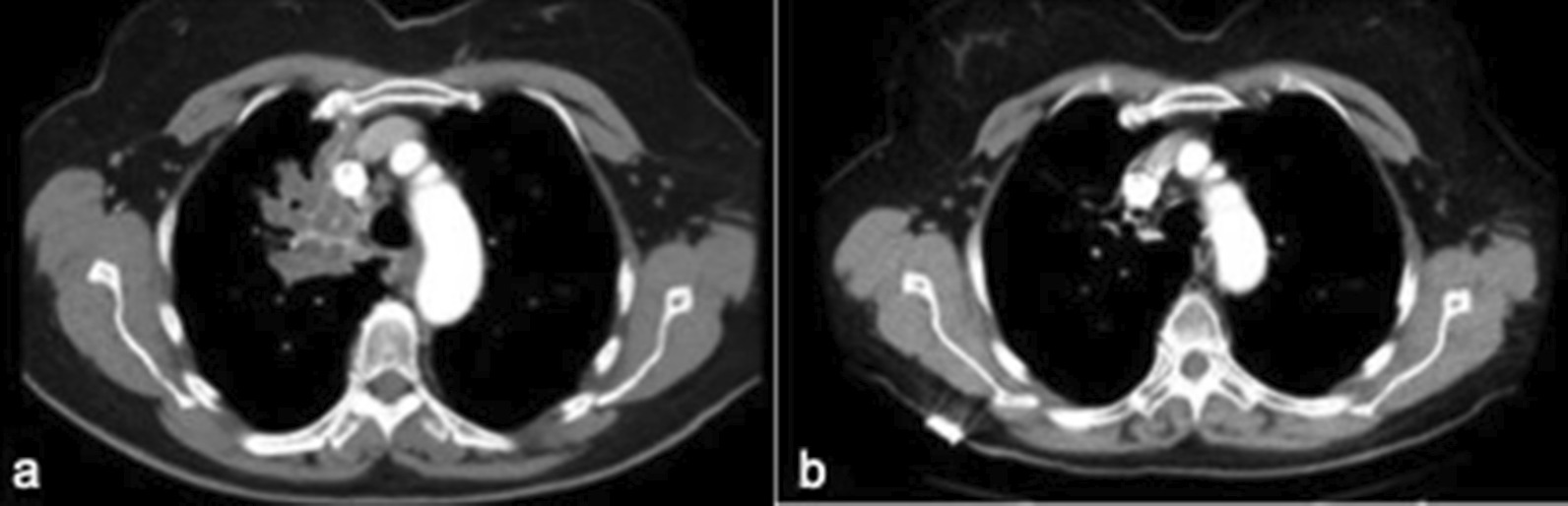
CT chest, axial, comparing multi-loculated lesion in right upper lobe before (**a**) and after treatment (**b**)

**Fig. 2:**
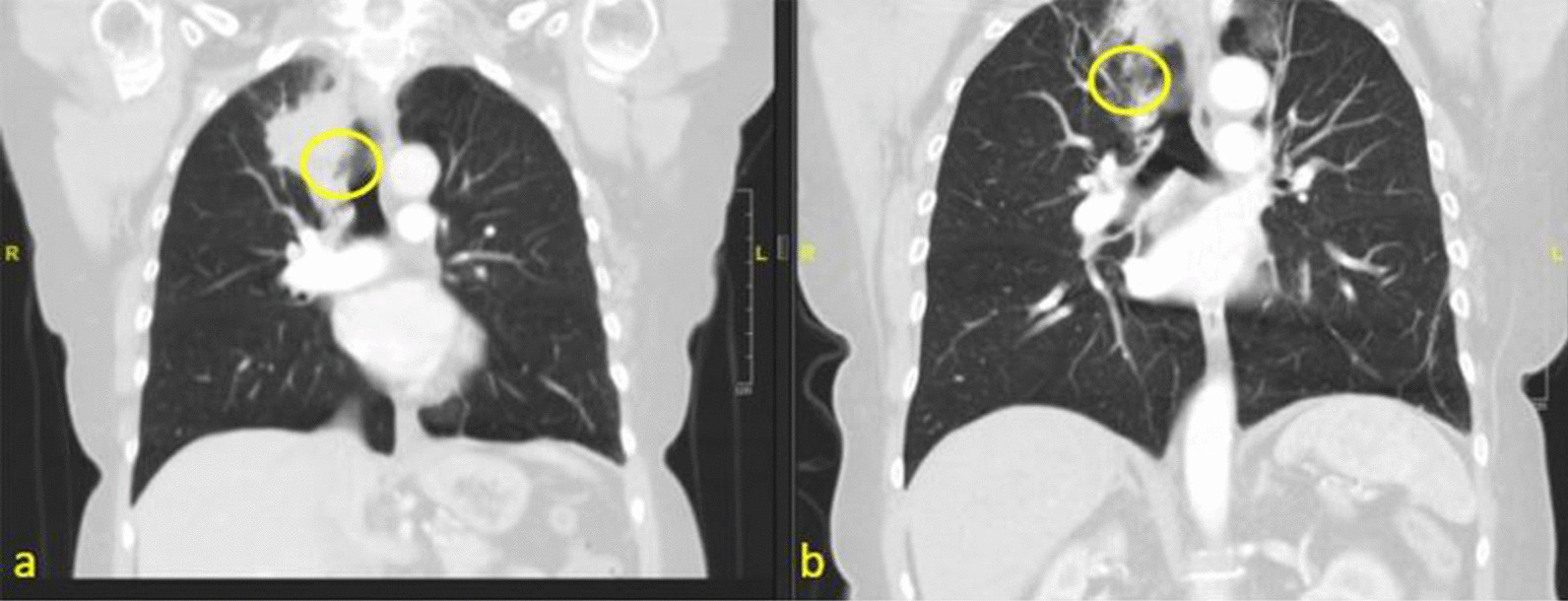
CT scan, coronal, comparing obstructed accessory bronchus before treatment (**a**) and patent bronchus after treatment (**b**)

**Fig. 3:**
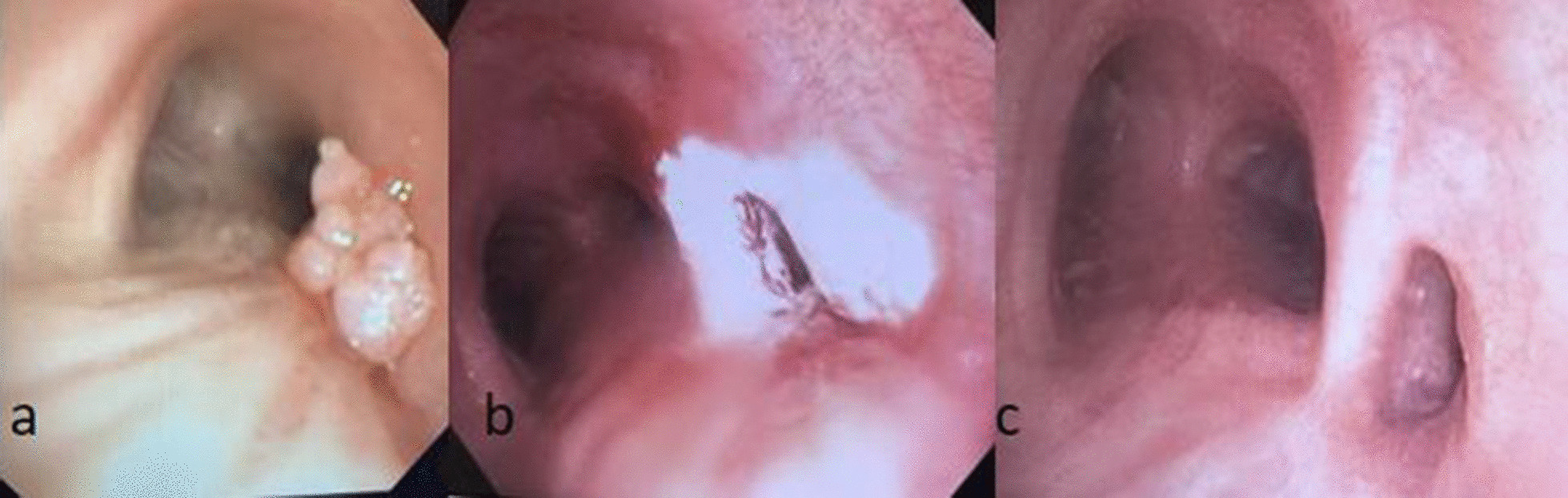
Image taken during bronchoscopy showing large papilloma obstructing accessory bronchus (**a**), application of cryotherapy to area (**b**), and patent airway after most recent cryotherapy treatment (**c**)

71-year-old lifelong nonsmoker with history of asthma and rheumatoid arthritis in good state of health presented to her pulmonologist with one year of cough, fevers, night sweats, and intermittent hemoptysis. She underwent CT scan of the chest which demonstrated a multiloculated density in the RUL (Figs. 1a and 2a). Diagnostic bronchoscopy revealed an obstructing mass extending into the tracheal lumen and involving an accessory bronchus to the RUL with no other airways involved. This mass was not apparent on her pre-operative imaging. The mass was extirpated with biopsy forceps which resulted in drainage of purulent material and subsequent visualization and patency of the accessory bronchus to the RUL. She completed a full course of oral antibiotics and one month later, she presented for scheduled surveillance bronchoscopy and cryotherapy. Residual papilloma within the accessory bronchus was noted and treated with cryotherapy using three five second treatments of liquid nitrogen (Fig. 3b). The patient had two subsequent treatments over the time span of 9 months, roughly three months between each treatment cycle, and has continued to undergo surveillance bronchoscopy. Now almost two years out from therapy, the patient has experienced complete resolution of her dyspnea and cough as a result of the cryotherapy treatments. She has maintained durable patency of her accessory bronchus evident on post treatment imaging (Figs. 1b and 2b) and surveillance bronchoscopy (Fig. 3c). Post treatment imaging also confirms resolution of post-obstructive atelectasis involving the right upper lobe. The unique presentation of an accessory bronchus in conjunction with papillomatous growth adds further complexity to the literature on this unique disease process.

## Discussion

There have been several case reports describing success of various methods to treat recurrent respiratory papilloma as well as some retrospective studies. However, given the rarity of this condition, there have been no randomized control trials comparing efficacy of different treatment modalities. In our case, the degree of obstruction necessitated endoscopic excision followed by treating remaining tissue with cryotherapy to maintain patency of the airway. While cryotherapy was our treatment method of choice, there are several available options for ablating the remaining tissue endoscopically including laser ablation and electrocautery, but there is limited data comparing these modalities. Given the low risk for malignant transformation of airway papillomas, major lung resection with potential need for complex airway reconstruction may not be justified if effective, endoscopic treatment such as cryotherapy proves to be durable. The role of post treatment surveillance with outpatient bronchoscopy is well tolerated and repeat treatments could be offered as needed for local recurrence.

Regarding surgical management particularly in this patient, cryotherapy has provided a lung sparing treatment modality that otherwise would have necessitated upper lobectomy with partial tracheal resection and tracheoplasty to achieve negative margins. Use of cryotherapy is especially relevant in patients with compromised lung function who cannot tolerate lung resection. While our patient didn’t have compromised lung function at baseline, her unique anatomy made surgical excision more difficult, and cryotherapy offered a preferrable alternative.

There have been several articles published on adjuvant medical therapy, particularly bevacizumab, in patients with recurrent respiratory papillomatosis [7]. Research regarding intralesional or systemic application of these medications is ongoing, though promising preliminary results have been published [6–8]. Furthermore, a recent review article on biologic therapy for this disease remarked that Gardasil HPV vaccine not only acts as an adjuvant treatment modality, but also prevents this illness in a meaningful way that could reduce occurrence of this disease in most of the developing world [8]. Future investigation is needed, but our case report contributes to the growing body of data on successful treatment of tracheal papillomatosis and is the only publication we have identified referencing this disease process in a tracheal bronchus.

## Data Availability

Not applicable to this study.
